# Comparative Study of the Efficacy of Lime Essential Oil Shampoo Versus 2% Miconazole/Chlorhexidine Combination Shampoo for the Treatment of Dermatophytosis in Client-Owned Cats

**DOI:** 10.3390/vetsci13010052

**Published:** 2026-01-07

**Authors:** Thapanee Chuenngam, Suttiwee Chermprapai

**Affiliations:** 1 Dermatology Center, Kasetsart University Veterinary Teaching Hospital, Faculty of Veterinary Medicine, Kasetsart University, Bangkok 10900, Thailand; fvettnc@ku.ac.th; 2 Department of Companion Animal Clinical Sciences, Faculty of Veterinary Medicine, Kasetsart University, Bangkok 10900, Thailand

**Keywords:** cats, lime essential oil shampoo, 2% miconazole/chlorhexidine, itraconazole, *Microsporum canis*

## Abstract

We undertook a preliminary clinical study to compare the efficacy of lime essential oil shampoo with a conventional 2% miconazole/chlorhexidine formulation, both in combination with pulse oral itraconazole, in cats with dermatophytosis caused by *M. canis*. Sixteen affected cats were randomly assigned to receive either lime essential oil shampoo or a 2% miconazole/chlorhexidine formulation. All cats were bathed twice weekly and received itraconazole (5 mg/kg once daily) using a pulse regimen (1 week for every 2 weeks) for 56 days. Clinical assessments, including cytological, direct hair examinations, Wood’s lamp evaluation, total skin lesion score (TLS), and fungal pathogen score (FPS), were performed on days 0, 28, 42, and 56. Hematological and biochemical analyses were conducted on days 0, 28, and 56. By day 56, no significant differences were observed between groups in the cytological, direct hair examination, or Wood’s lamp results. Both groups had significant reductions in TLS and FPS on days 28, 42, and 56 compared to day 0 (*p* < 0.05). No cutaneous or systemic adverse effects were observed. These findings suggest that lime essential oil shampoo has clinical efficacy comparable to the conventional formulation and may represent a safe natural alternative for the topical management of feline dermatophytosis.

## Simple Summary

*Microsporum canis* (*M. canis*) is the most common cause of dermatophytosis in cats and can also be transmitted to humans. In most cases, infected cats develop circular areas of hair loss, scaling, or crusting, and the condition can easily spread through direct contact or contaminated environments. The standard treatment includes the use of topical antifungal shampoos combined with oral antifungal medications to eliminate fungal spores from the hair coat and skin. Conventional products, such as miconazole and chlorhexidine shampoos, are widely used and effective; however, there is increasing interest in natural plant-based alternatives that may offer similar benefits with fewer chemical ingredients. Lime essential oil has demonstrated antifungal properties in laboratory studies, suggesting its potential use as a natural topical therapy. This study explored the application of lime essential oil shampoo as a novel treatment option for feline dermatophytosis.

## 1. Introduction

*Microsporum canis* is the most common cause of dermatophytosis in cats [1,2,3,4,5,6,7,8,9,10], which is transmitted through direct contact with other infected animals and humans [1,4,5,9,11]. Some cats require several months to self-limit [1,6,8], while those with the clinical signs of dermatophytosis typically present with concurrent skin or systemic disease and/or physiological stress, resulting in multifocal and diffuse lesions [1,8,12,13]. There are a variety of skin lesions of keratinized structures, including alopecia, papules, scales, crusts, follicular cast, hyperpigmentation, and abnormality of the nail structure [1,2,8,10,12,13]. The standard treatment for dermatophytosis includes topical antifungal treatment to eliminate the spreading of fungal arthrospores and to limit reinfection and new infection; a combination of 2% miconazole/2% chlorhexidine gluconate is currently recommended as an effective topical therapy [1,7,8,13,14,15]. Systemic antifungal medications (to eliminate active fungal infection and proliferation on the skin and hair) and non-compounded itraconazole and terbinafine are the most effective and safe medications for dermatophytosis. Environmental control is used to prevent fomite contamination and reinfection from the environment [1,3,6,8,9,15,16].

Recently, there has been growing interest in the use of natural and plant-based therapies for the treatment of dermatophytosis in cats [1,16]. Several essential oils—such as *Thymus serpyllum*, *Origanum vulgare*, and *Rosmarinus officinalis*—have shown promising antifungal activity against *M. canis* in vitro and in vivo [1,9,16,17,18,19,20]. However, while lime (*Citrus aurantifolia*) essential oil has been documented to contain bioactive compounds, such as limonene, which exhibit antifungal effects through the disruption of fungal cell wall integrity in vitro, and possess antioxidant, antimicrobial, and skin emollient properties in vitro [18,21,22,23,24,25,26,27], its clinical efficacy against *M. canis* in cats has not yet been evaluated. Lime is widely cultivated in Southeast Asia and is an economically important crop in Thailand [28,29]; therefore, it represents a readily available candidate for natural-based topical therapies.

Thus, the aim of the current study was to assess the therapeutic efficacy of a lime essential oil-based shampoo in the treatment of feline dermatophytosis caused by *M. canis* and to compare its clinical outcomes with a commercially available 2% miconazole/2% chlorhexidine gluconate shampoo, both used in combination with pulse oral itraconazole, in client-owned cats. In addition, the potential of lime essential oil as a natural alternative for topical antifungal therapy in veterinary dermatology was explored.

## 2. Materials and Methods

### 2.1. Ethics

This study was approved by the Institute Animal Care and Use Committee of Kasetsart University, Bangkok, Thailand (approved ID. ACKU67-VET-012). The experimental purpose and process of the study were explained to each cat owner, who signed a consent form at the onset of the study.

### 2.2. Animals and Inclusion and Exclusion Criteria

The sample consisted of sixteen client-owned cats aged between 6 months and 11 years, presenting with focal or generalized skin lesions such as alopecia, scale, crust, and follicular cast. The number of enrolled cats was determined based on a power analysis using effect sizes (α = 0.05, power = 80%), reported from the previous open-field studies evaluating topical antifungal shampoos in feline dermatophytosis [15,16], and the availability of client-owned animals that met the inclusion criteria during the study period. Ectoparasite infestation was ruled out via direct examination and routine ectoparasite control. Bacterial infection was ruled out via skin cytology.

None of the cats had received topical or systemic antifungal treatment within the previous 6 weeks. Cats that had received topical or systemic antifungal treatment within the previous 6 weeks and/or had developed systemic disease unrelated to this study were excluded. The diagnosis of dermatophytosis was confirmed by at least one of the following criteria: strongly fluorescent hairs under Wood’s lamp examination, the presence of arthroconidia and/or hyphae observed in skin cytology, or detection of ectothrix fungal spores and/or hyphae on keratinized hair shafts via direct hair examination. The presence of any one of these diagnostic findings along with a positive culture on day 0 (D0) was considered sufficient for inclusion in the study.

#### 2.2.1. Wood’s Lamp Examination

Wood’s lamp examination was performed using an ultraviolet (UV) light device that emits long-wave UV radiation in the range of approximately 320–400 nm. Infected hairs were identified by the presence of a characteristic bright apple-green fluorescence, which confirms *M. canis* infection.

#### 2.2.2. Cytological Evaluation

Cytological evaluation of the affected skin lesions was performed using the cotton swab technique or impression smear. The collected samples were stained with Diff-Quik (R.C.M. Supplies Co., Ltd.; Bangkok, Thailand) and examined under oil immersion at 1000× magnification. Arthroconidia and/or hyphae were identified based on their characteristic morphological features.

#### 2.2.3. Direct Hair Examination

Suspected *M. canis*-infected hairs were collected from skin lesions using sterile forceps, placed on a glass slide with mineral oil, covered with a coverslip, and examined under a microscope. The presence of ectothrix spores and/or hyphae on the keratinized hair shaft was considered confirmatory for infection.

### 2.3. Study Design and Protocol

A randomized single-blinded clinical trial over 56 days was conducted from July 2024 to March 2025. The client-owned cats with skin lesions of *M. canis* infection were divided equally into two groups using simple random sampling: group A, lime essential oil shampoo; group B, 2% miconazole/2% chlorhexidine gluconate (Malaseb^®^, Ensign Laboratories PTY Ltd., Mulgrave, Australia) shampoo. In this single-blinded design, the owners were blinded to the treatment allocation, and both shampoos were dispensed in identical unlabeled containers to minimize the recognition based on product appearance. The cats in both groups were washed using the same frequency (twice a week), with 10 min of contact time, followed by rinsing, throughout the study period. All cats in both groups received a pulse administration of 5 mg/kg once daily of oral itraconazole (Sporna, S. Charoen Bhaesaj Trading Co., Ltd.; Bangkok, Thailand) for 1 week every 2 weeks over the study period. All pharmaceutical products used in this study were obtained from a single production batch to ensure consistency in quality and efficacy. In addition, the owners were instructed to disinfect the environment and related devices at least twice weekly using a 1:10 diluted bleach solution (Haiter^®^; Kao Industrial (Thailand) Co., Ltd.; Bangkok, Thailand, or Hilyte^®^; Vet Planet Co., Ltd.; Bangkok, Thailand), with a 10-min contact time. The treatment took place at home, with follow-up on D28, D42, and D56. The skin lesion score, Wood’s lamp examination, skin cytological examination, fungal culture, and CFU/plate for pathogen score were recorded at each visit; blood analyses were collected during the first visit (D0) and, subsequently, on D28 and D56 after receiving medication, to monitor the hematological and biochemical changes after treatment. The primary outcomes were defined as decreases in the total skin lesion score (TLS) and fungal pathogen score (FPS). Clinical cure was defined as a TLS of 3 out of 12, and mycological cure was defined as two consecutive negative fungal cultures. The absence of undesirable cutaneous or systemic adverse effects related to the study treatments was defined as a secondary outcome.

#### 2.3.1. Fungal Culture

**Figure 1:**
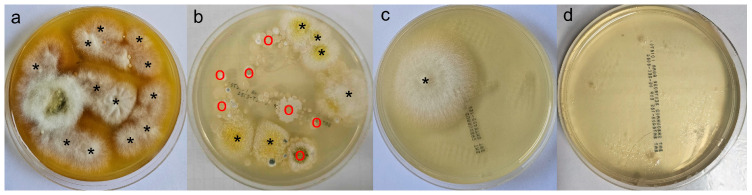
Examples of culture plates obtained, graded (P1–P4 or no growth) according to the pathogen score based on the CFU count per plate: (**a**) P3, ≥10 CFUs/plate; (**b**) P2, 5–9 CFUs/plate; (**c**) P1, 1–4 CFUs/plate; and (**d**) no growth. Colony identifiers: * *Microsporum canis*; o, nondermatophytic mould.

Fungal culture was used to evaluate the fungal load by counting colony-forming units (CFUs). Hair and skin samples were collected using the modified McKenzie toothbrush technique. In brief, a sterile toothbrush was gently rubbed 20 times over the lesions on the body of each cat; then, the bristles were gently pressed onto five sites on a 90 mm diameter Sabouraud dextrose agar plate. This procedure was performed by the same person to minimize human error. Plates were incubated at 25 °C and examined during 7–21 days. Fungal colonies were identified to the species level based on their morphology and microscopic features and graded using a pathogen score based on a CFU count per plate, using the following pathogen score classification: P1, 1–4 CFU/plate; P2, 5–9 CFU/plate; P3, ≥10 CFU/plate; and no growth (Figure 1). This method was used to evaluate changes in the clinical response to treatment on D28, D42, and D56, compared to D0.

#### 2.3.2. Skin Lesion Score

At each visit, a skin lesion score was assigned using physical examination of the skin lesions to evaluate the clinical improvement. The skin lesion score was divided into 3 groups, as shown in Table 1, describing the ease of epilation, degree of seborrhea, and the extent of primary lesions [15,16]. The score in each group was in the range 3–12, recorded on D0, D28, D42, and D56.

### 2.4. Lime Essential Oil Shampoo

The shampoo used in group A was developed by InnoHERB: Expert Centre of Innovative Herbal Products, Thailand Institute of Scientific and Technological Research, Bangkok, Thailand. It contained lime essential oil at a concentration of 1% (*v*/*v*), which includes bioactive compounds such as limonene that have been reported to exert antifungal effects through the disruption of fungal cell wall integrity [21,24,25,28], along with other ingredients such as allantoin, polysorbate 20, D-panthenol, and phenoxyethanol. Prior to its application, the shampoo underwent comprehensive safety evaluations approved by the Institute Animal Care and Use Committee of the Thailand Institute of Scientific and Technological Research (approved No. T-66008). An acute oral toxicity test in rats revealed that the product was in Category 5 or unclassified under the Globally Harmonized System of Classification and Labelling of Chemicals (GHS), with a median lethal dose (LD_50_) value exceeding 5000 mg/kg body weight. Similarly, the dermal toxicity testing indicated that the product was also in Category 5 or unclassified, with an LD_50_ range of 2000–5000 mg/kg body weight. Based on these findings, the lime essential oil-based shampoo could be considered safe for use in companion animals, including scenarios involving accidental ingestion or self-grooming of the treated hair coat.

### 2.5. Adverse Effects

On D0, D28, and D56, blood samples were collected to evaluate the health status and to determine any adverse effects of treatment based on the analysis of hematological and serum biochemical parameters. Blood samples were collected from the jugular vein or median saphenous vein in ethylenediaminetetraacetic acid tubes and serum-separate tubes. Hematological analysis was carried out for hematocrit (HCT), plasma protein (PP), white blood cell count (WBC), and platelets, using an automated hematology analyzer (XN-1000 Vet, Sysmex, Kobe, Japan). The serum biochemical parameters, consisting of blood urea nitrogen (BUN), creatinine (CREA), alanine transaminase (ALT), aspartate aminotransferase (AST), total protein (TP), and albumin (ALB), were analyzed using an automated chemistry analyzer (iLab Taurus, Instrumentation Laboratory, Werfen Group, Barcelona, Spain) and compared with those on D0 after receiving itraconazole and topical agents. Cutaneous safety was assessed clinically through serial physical examinations and owner-reported observations of skin irritation, pruritus, and inflammation.

### 2.6. Statistical Methods

The data recorded were entered into an Excel spreadsheet and analyzed using Jamovi (The jamovi project (2025). jamovi (Version 2.6) [Computer Software]. Retrieved from https://www.jamovi.org accessed on 1 September 2025) and Microsoft Excel 2019 (MS OFFICE; Microsoft, Redmond, WA, USA). The demographic data on animals (age, sex, and breed) were presented as median and interquartile ranges, as well as analyzed descriptively. Fisher’s exact test was used to compare treatment groups among cytology, direct hair examination, and Wood’s lamp examination. The Shapiro–Wilk test was performed to assess the normality of data. To compare the total skin lesion scores (TLSs) and fungal pathogen scores (FPSs) on D0, D28, D42, and D56 within the same group, paired Student’s *t*-test analysis was performed when the data were normally distributed, while the Wilcoxon signed-rank test was performed when the data were not normally distributed. The Mann–Whitney U test was used to compare the TLS and FPS values between the two treatment groups. Statistical significance was defined as *p* < 0.05.

## 3. Results

Of the 16 cats, 8 were in group A (lime essential oil shampoo) and 8 in group B (Malaseb^®^). The average age of the cats was 4.9 years (range 1.7–11 years) and 3.4 years (range 2–9 years) in groups A and B, respectively. The breeds consisted of seven Domestic Short Hair (43.75%), two Exotic Short Hair (12.5%), two Exotic Long Hair (12.5%), two Persians (12.5%), two Scottish folds (12.5%), and one Longhair Mixed Breed (6.25%). There were five short-haired coat cats and three long-haired coat cats in each group. In group A, three of eight cats presented with generalized lesions, and four of eight originated from multi-cat households, whereas in group B, four of eight cats had generalized lesions, and two of eight originated from multi-cat households.

### 3.1. Cytology, Direct Hair Examination, and Wood’s Lamp Examination

The distribution of demographic information in each week is presented in Table 2. On D0, arthroconidia and/or fungal hyphae were identified in all cats in both groups. At subsequent evaluations (D28, D42, and D56), a reduction in fungal elements was observed. In group A, arthrospores were detected in two cats on D28, one on D42, and two on D56. In group B, arthrospores were observed in two cats on D28, one on D42, and one on D56. At baseline (D0) and on D56, there were no statistically significant differences between the treatment groups in the proportion of positive cases based on cytological examination, direct hair examination, or Wood’s lamp examination (Fisher’s exact test, two-sided; D0: cytology, *p* = 1.000; direct hair examination, *p* = 1.000; Wood’s lamp examination, *p* = 1.000; D56: cytology, *p* = 1.000; direct hair examination, *p* = 1.000; Wood’s lamp examination, *p* = 0.57), as shown in Table 3.

### 3.2. Total Skin Lesion Score

**Figure 2:**
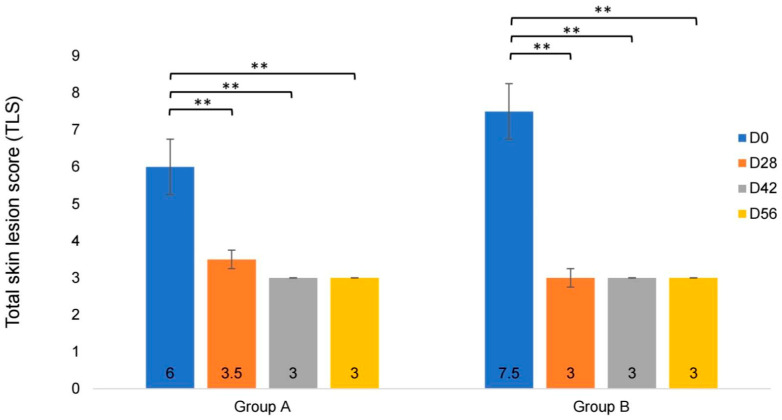
Changes in total skin lesion score (TLS), expressed as the median and SD range, on D0, D28, D42, and D56 of treatment with lime essential shampoo (group A) and 2% miconazole and 2% chlorhexidine shampoo (group B), showing a significant decrease in TLS within group at every point of study (** *p* < 0.01).

The median values of the TLS for both groups are presented in Table 4 and Figure 2. For the within-group comparisons based on D0, there were significant decreases in TLS on D28, D42, and D56 for both groups (*p* < 0.01), as shown in Figure 2. However, there were no significant differences between the lime shampoo and Malaseb^®^ groups at any time point (D0: *p* = 0.064; D28: *p* = 0.858; D42: *p* = 1.000; D56: *p* = 0.700).

### 3.3. Fungal Pathogen Score

**Figure 3:**
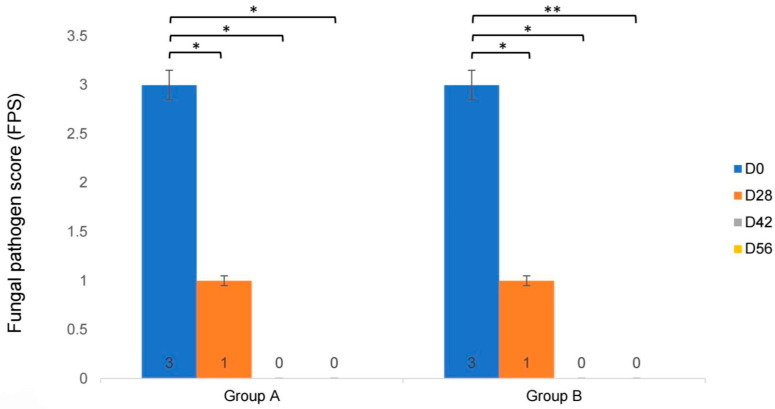
Changes in fungal pathogen score (FPS), expressed as the median and SD range, on D0, D28, D42, and D 56 of treatment with lime essential oil shampoo (group A) and 2% miconazole and 2% chlorhexidine shampoo (group B), showing a significant decrease in FPS within group at every point of study (* *p* < 0.05, ** *p* < 0.01).

The median FPS values for both groups are presented in Table 5 and Figure 3. Compared to D0, both groups showed a significant decrease in FPS on D28, D42, and D56 (group A, *p* = 0.012, *p* = 0.013, and *p* = 0.012, respectively; group B, *p* = 0.021, *p* = 0.015, and *p* = 0.008, respectively), as shown in Figure 3. No significant differences were observed between the lime shampoo and Malaseb^®^ groups at any time point (D0, *p* = 0.075; D28, *p* = 0.700; D42, *p* = 1.000; D56, *p* = 0.369).

### 3.4. Clinical Cure and Mycological Cure

Clinical cure and mycological cure were observed in six of eight and five of eight cats in Group A and in seven of eight and six of eight cats in Group B, respectively.

### 3.5. Adverse Effects

Blood analysis was performed on D0, D28, and D56. In both treatment groups, no significant differences were observed in the PP, PLT count, ALT, AST, BUN, CREA, TP, and ALB levels on D0, D28, or D56 (Table 6). All hematological and serum biochemical parameters were within the reference ranges at baseline (D0) in both groups.

In the lime essential oil shampoo group, the HCT levels significantly increased on D56 [median 39.1% (IQR 27.9–45.2), *p* = 0.008], compared to D0 [median 36.3% (IQR 26.1–40.4)]. In the Malaseb^®^ group, the HCT levels also significantly increased on D28 [median 36.4% (IQR 30.2–40.4), *p* = 0.028] and D56 [median 36.9% (IQR 27.5–42.4), *p* = 0.026], compared to D0 [median 32.8% (IQR 29.6–41.8)]. The WBC count significantly decreased within the reference range on D28 in the Malaseb^®^ group [median 11.4 × 10^3^/μL (IQR 5.09–16.6), *p* = 0.021], compared to D0 [median 13.3 × 10^3^/μL (IQR 6.49–18.6)]. There were no significant changes in the WBC count in the lime essential oil shampoo group. In addition, there were no reported adverse clinical effects, including hypersalivation, vomiting, diarrhea, anorexia, skin irritation, pruritus, and inflammation in any of the cases.

**Table 6:** Hematological and serum biochemistry parameters on day 0 (D0), D28, and D56 of treatment with the lime essential oil shampoo (group A) and Malaseb shampoo (group B).

	GROUP A (*n* = 8)			GROUP B (*n* = 8)			Reference Range
	D0	D28	D56	D0	D28	D56	
HCT (%)	36.3 (26.1–40.4)	38.7 (29.7–41.5)	39.1 ** (27.9–45.2)	32.8 (29.6–41.8)	36.4 * (30.2–40.4)	36.9 ** (27.5–42.4)	30–45
PLT (×10^3^/uL)	292 (224–438)	270 (65–406)	261 (172–521)	347 (261–552)	401 (23–556)	350 (137–532)	300–800
WBC (×10^3^/uL)	8.73 (6.32–28.3)	7.57 (5.25–16.2)	8.09 (6.09–18.9)	13.3 (6.49–18.6)	11.4 * (5.09–16.6)	13 (3.74–16.3)	5.50–19.50
PP (g/dL)	7.8 (7.0–8.4)	7.6 (7.0–8.0)	7.9 (7.0–8.6)	7.1 (6.4–8.2)	7.3 (6.0–8.0)	7.8 (6.0–8.6)	6.0–8.0
BUN (mg/dL)	22.5 (7.8–30)	23 (8–31)	22.5 (15–31)	20 (16–28)	20 (14–27)	22 (15–32)	19–34
CREA (mg/dL)	1.61 (1.06–1.88)	1.54 (0.98–2.1)	1.6 (1.02–1.92)	1.06 (0.59–1.38)	0.98 (0.78–1.41)	1.14 (0.72–1.53)	0.9–2.2
ALT (U/L)	47 (34–87)	48.5 (32–179)	47.5 (33–94)	59 (19–144)	47 (27–116)	43 (27–96)	25–97
AST (U/L)	27 (12–45)	23.5 (12–57)	27.5 (14–42)	28 (17–46)	24 (12–41)	25.5 (12–36)	7–38
TP (g/dL)	7.1 (7.0–8.0)	7.15 (6.6–7.6)	7.3 (6.7–8.0)	6.7 (5.7–8.1)	6.95 (5.8–8.0)	7.2 (6.3–7.8)	6.0–7.96
ALB (g/dL)	3.35 (2.7–3.8)	3.4 (2.8–3.5)	3.4 (2.7–3.7)	3.15 (2.3–3.4)	3.15 (2.4–4.0)	3.3 (2.5–3.7)	2.8–3.9

Note: All data are presented as the median and interquartile range. Abbreviations: ALB, albumin; ALT, alanine aminotransferase; AST, aspartate aminotransferase; BUN, blood urea nitrogen; CREA, creatinine; HCT, hematocrit; PLT, blood platelet count; PP, plasma protein; TP, total protein; WBC, white blood cell count. * *p* < 0.05, compared with D0. ** *p* < 0.01, compared with D0.

## 4. Discussion

In cats with dermatophytosis, topical therapy is a crucial component of management strategies aimed at reducing the infectious, contagious, and zoonotic risks by disinfecting the hair coat and minimizing the shedding and dissemination of infective material into the environment [1,3,7,14,16]. Recommended topical agents include lime sulfur, enilconazole, or a combination of 2% miconazole and chlorhexidine shampoo, applied twice weekly [1,7,13,16]. A 2% miconazole/chlorhexidine combination shampoo is widely available and commonly used due to its proven efficacy [3,11,14,15,16].

In recent years, interest in natural plant-derived substances has increased, with various essential oils showing effectiveness against fungal infections in both human and veterinary medicine [16,17,18,21,22,23,24,28]. Lime essential oil (*Citrus aurantifolia*) has been identified in other studies as having notable antifungal activity, as well as beneficial skin emollient effects [17,18,21,22,23,24,25,26,28]. While its use in feline dermatophytosis has not yet been investigated, the use of lime essential oil in a natural-based shampoo represents a novel therapeutic approach and may offer a promising alternative for the topical management of dermatophytosis in cats.

This preliminary study evaluated the efficacy of lime essential oil shampoo compared to a conventional formulation containing 2% miconazole and 2% chlorhexidine (Malaseb^®^), in combination with pulse oral itraconazole for both groups, for the treatment of feline dermatophytosis. The clinical assessments were based on the total lesion score (TLS) and fungal pathogen score (FPS) values over a 56-day period. Both treatment groups had significant reductions in TLS and FPS values over time, with no statistically significant differences observed between the two groups at any time point. Additionally, there were no significant differences in cytology, direct hair examination, or Wood’s lamp evaluation, suggesting that lime essential oil shampoo has comparable clinical and antifungal efficacy and supporting its potential role as an alternative topical treatment for feline dermatophytosis. The clinical responses to the topical therapy can be attributed to the antifungal properties and physical cleansing of the topical formulations used.

The limitations of the current study included the small number of cats in each treatment group, due to challenges in recruiting pet cats that met the inclusion criteria, which may have limited the statistical power, and the use of multiple comparisons across outcomes and time points, which may increase the risk of a Type I error; therefore, non-significant findings should be interpreted with caution. Additional limitations included the lack of a control group without topical and systemic therapy due to ethical concerns in a client-owned cat study; the wide age range of the enrolled cats; the potential influence of age-related differences in immune function; the relatively short 56-day study period; the lack of environmental culture monitoring; and the potential for passive fungal contamination. Although owners were instructed to disinfect the environment and related devices using a 1:10 diluted bleach solution (Haiter^®^; Kao Industrial (Thailand) Co., Ltd.; Bangkok, Thailand, or Hilyte^®^; Vet Planet Co., Ltd.; Bangkok, Thailand), environmental contamination of the hair coat could not be completely ruled out.

Differences in lesion distribution and environmental exposure may have influenced individual treatment responses. The higher proportion of multi-cat households in group A, with close contact with other potentially asymptomatic cats, suggests possible subclinical infection or recontamination, while group B had a larger number of cats with generalized lesions; these different factors in each group might have contributed to differences in the TLS and FPS values and treatment duration.

In the study cats, on D56, some cats in both groups still showed positive results for arthroconidia on cytological examination, Wood’s lamp examination, and fungal culture. Several factors may have contributed to this outcome, including insufficient environmental management within individual households, the seasonal timing of the study period with high humidity, and the severity of infection [1,30]. In group A, one Exotic Shorthair cat with generalized lesions remained positive across all diagnostic methods. This cat resided in a multi-cat household and had close contact with other suspected asymptomatic cats, suggesting possible subclinical infection or recontamination. The owner had been advised to clip the cat’s hair coat and perform weekly environmental cleaning; however, they were unable to comply, due to time constraints. Another case involved a Long-Haired Persian cat, a breed predisposed to dermatophytosis [1,12,30], and an individual that habitually rested in the litter box and moist restroom areas, likely contributing to delayed recovery due to persistently damp skin. In group B, one Longhair Mixed Breed cat with a severe fungal infection remained positive for fungal culture on D56, suggesting that the duration of the treatment may have been insufficient and that a longer treatment and recovery period may be necessary in cases with a heavy fungal infection.

The safety profile of the lime essential oil shampoo was carefully evaluated, as this was a novel alternative topical agent. Hematological and serum biochemical parameters were monitored throughout the study, with no abnormalities detected. No adverse dermatological reactions, such as increased erythema, pruritus, scaling, or skin irritation, were observed in either treatment group throughout the study period. In addition, no adverse clinical signs—such as vomiting, diarrhea, or anorexia—were reported in any of the treated cats. Furthermore, owners reported satisfaction with the product’s efficacy and pleasant scent, with no issues noted regarding its use.

Therefore, these preliminary study findings indicate that lime essential oil shampoo is comparable in efficacy with a conventional 2% miconazole/chlorhexidine formulation for the topical treatment of dermatophytosis in cats. This natural-based alternative may be particularly beneficial in settings where conventional treatments are unavailable, poorly tolerated, or when the owner prefers botanical options. Further studies are warranted with larger sample sizes, extended treatment durations, and environmental fungal monitoring to confirm the long-term efficacy, assess the recurrence rates, and better understand the role of lime essential oil in antifungal therapy.

## 5. Conclusions

This preliminary study introduced a novel topical treatment for feline dermatophytosis by comparing the efficacy of lime essential oil shampoo to a conventional 2% miconazole/chlorhexidine formulation, both in combination with pulse oral itraconazole. The results suggest comparable clinical and antifungal effectiveness without undesirable adverse effects; however, due to the limited sample size and variability between cases, these findings should be interpreted with caution. Further controlled studies with larger sample sizes are warranted to confirm the efficacy and safety.

## Figures and Tables

**Table 1:** Clinical score data of the skin lesion score to evaluate clinical improvement.

Score	Skin Lesion		
	Ease of Epilation	Degree of Seborrhea	Extent of Primary Lesion
**1**	Within normal limits	None	None
**2**	Mild but excessive	Mild	Single small area
**3**	Moderate	Moderate	More than one small area
**4**	Severe and extensive	Severe	Extensive lesions

**Table 2:** Demographics of positive cases based on cytological examination, direct hair examination, and Wood’s lamp (*n* = 8, each group) on day 0 (D0), D28, D42, and D56.

		Group A (%)	Group B (%)
**Cytology**	D0	8/8 (100%)	8/8 (100%)
	D28	2/8 (25%)	2/8 (25%)
	D42	1/8 (12.5%)	1/8 (12.5%)
	D56	2/8 (25%)	1/8 (12.5%)
**Direct hair**	D0	8/8 (100%)	7/8 (87.5%)
	D28	0/8 (0%)	1/8 (12.5%)
	D42	1/8 (12.5%)	0/8 (0%)
	D56	1/8 (12.5%)	0/8 (0%)
**Wood’s lamp**	D0	8/8 (100%)	8/8 (100%)
	D28	4/8 (50%)	4/8 (50%)
	D42	2/8 (25%)	1/8 (12.5%)
	D56	3/8 (37.5%)	1/8 (12.5%)

**Table 3:** Results of positive cases based on cytological examination, direct hair examination, and Wood’s lamp on D56 (*n* = 8, each group).

	Cytology (%)		Direct Hair Examination (%)		Wood’s Lamp (%)	
**Evaluation Day**	D0	D56	D0	D56	D0	56
**Group A**	8/8 (100%)	2/8 (25%)	8/8 (100%)	1/8 (12.5%)	8/8 (100%)	3/8 (37.5%)
**Group B**	8/8 (100%)	1/8 (12.5%)	8/8 (100%)	0/8 (0%)	7/8 (87.5%)	1/8 (12.5%)
**Fisher’s Exact Test** **Exact Sig. (2-sided)** ***p*-value**	1	1	1	1	1	0.57

**Table 4:** Median of total skin lesion score (range 3–12), for groups A and B, assessed on D0, D28, D42, and D56 of treatment.

	Group A		Group B		Mann–Whitney U *p*-Value
Evaluation Day	Median	Range	Median	Range	
**D0**	6.0	5–8	7.5	6–12	0.064
**D28**	3.5	3–4	3.0	3–8	0.858
**D42**	3.0	3–4	3.0	3–6	1.000
**D56**	3.0	3–4	3.0	3–5	0.700

**Table 5:** Median of fungal pathogen score, (range 0–3) for groups A and B, assessed on D0, D28, D42, and D56 of treatment.

	Group A		Group B		Mann–Whitney U *p*-Value
Evaluation Day	Median	Range	Median	Range	
**0**	3	2–3	3	3–3	0.075
**28**	1	0–2	1	0–3	0.700
**42**	0	0–2	0	0–3	1.000
**56**	0	0–2	0	0–2	0.369

## Data Availability

The original contributions presented in this study are included in the article. Further inquiries can be directed to the corresponding author.

## Abbreviations

The following abbreviations are used in this manuscript:

*M. canis*	*Microsporum canis*
TLS	Total skin lesion score
FPS	Fungal pathogen score
D	Day
